# “I have such a hard time hitting myself, I thought it’d be easier”: perspectives of hospitalized patients on injecting drugs into vascular access devices

**DOI:** 10.1186/s12954-022-00637-1

**Published:** 2022-05-26

**Authors:** Hannah L. Brooks, Ginetta Salvalaggio, Bernadette Pauly, Kathryn Dong, Tania Bubela, Marliss Taylor, Elaine Hyshka

**Affiliations:** 1 School of Public Health, University of Alberta, 3-300 Edmonton Clinic Health Academy, 11405 - 87 Ave NW, Edmonton, AB T6G 1C9 Canada; 2 Inner City Health and Wellness Program, Royal Alexandra Hospital, B811 Women’s Centre, 10240 Kingsway Avenue, Edmonton, AB T5H 3V9 Canada; 3 Department of Family Medicine, University of Alberta, 5-16 University Terrace, Edmonton, AB T6G 2T4 Canada; 4 Canadian Institute for Substance Use Research, University of Victoria, 2300 McKenzie Ave, Victoria, BC V8N 5M8 Canada; 5 Department of Emergency Medicine, University of Alberta, 790 University Terrace Building, 8303 – 112 Street, Edmonton, AB T6G 2T4 Canada; 6 Faculty of Health Sciences, Simon Fraser University, Blusson Hall, Room 11300, 8888 University Drive, Burnaby, BC V5A 1S6 Canada; 7 Streetworks, Boyle Street Community Services, 10116 105 Ave NW, Edmonton, AB T5H 0K2 Canada

**Keywords:** Vascular access devices, Substance-related disorders, Hospitalization, Harm reduction, Patient-centered care

## Abstract

**Background:**

Hospital patients who use drugs may require prolonged parenteral antimicrobial therapy administered through a vascular access device (VAD). Clinicians’ concerns that patients may inject drugs into these devices are well documented. However, the perspectives of patients on VAD injecting are not well described, hindering the development of informed clinical guidance. This study was conducted to elicit inpatient perspectives on the practice of injecting drugs into VADs and to propose strategies to reduce associated harms.

**Methods:**

Researchers conducted a focused ethnography and completed semi-structured interviews with 25 inpatients at a large tertiary hospital in Western Canada that experiences a high rate of drug-related presentations annually.

**Results:**

A few participants reported injecting into their VAD at least once, and nearly all had heard of the practice. The primary reason for injecting into a VAD was easier venous access since many participants had experienced significant vein damage from injection drug use. Several participants recognized the risks associated with injecting into VADs, and either refrained from the practice or took steps to maintain their devices while using them to inject drugs. Others were uncertain how the devices functioned and were unaware of potential harms.

**Conclusions:**

VADs are important for facilitating completion of parenteral antimicrobial therapy and for other medically necessary care. Prematurely discharging patients who inject into their VAD from hospital, or discontinuing or modifying therapy, results in inequitable access to health care for a structurally vulnerable patient population. Our findings demonstrate a need for healthcare provider education and non-stigmatizing clinical interventions to reduce potential harms associated with VAD injecting. Those interventions could include providing access to specialized pain and withdrawal management, opioid agonist treatment, and harm reduction services, including safer drug use education to reduce or prevent complications from injecting drugs into VADs.

## Background

Hospitalization rates due to infections associated with injection drug use (e.g., infective endocarditis, osteomyelitis, and skin, soft tissue, and pulmonary infections) are increasing in the USA and Canada [1–4]. These infections are caused by unsafe injection practices (e.g., injecting with non-sterile syringes) [1, 5], drug excipients in certain pharmaceutical drugs [6, 7], and/or fillers and other particulates in street-sourced drugs [8]. Treatment of these infections includes prolonged parenteral antimicrobial therapy administered through vascular access devices (VADs). VADs comprise various types of catheters inserted to access peripheral or central vessels (e.g., peripherally inserted central catheter (PICC), intravenous (IV) line) [9].

Due to the extended length of parenteral antimicrobial therapy, patients who do not use drugs are frequently discharged to home and treated as outpatients [10]. Conversely, people who inject drugs (PWID) often remain hospitalized for the duration of their treatment due to concerns that unstable housing or ongoing drug use may inhibit treatment adherence and that patients will inject drugs and/or diverted medications into these devices [9–11]. However, there is a lack of empirical evidence on the prevalence and health risks associated with injecting drugs into VADs among either outpatient or hospitalized PWID [12–15]. Nevertheless, clinical guidance in many jurisdictions advises inpatient hospitalization and close monitoring as the primary strategy to prevent VAD complications for this patient population [9–11].

Yet drug use can occur in hospital settings [16–20]. Cohort and structured surveys with PWID in Canada [16–18] and the USA [19, 20] report that between 30 and 50% of participants continued to use drugs while hospitalized. Injection drug use in hospital can occur for many reasons including uncontrolled pain and withdrawal symptoms or to manage symptoms of stress or anxiety [21, 22]. To circumvent discovery by hospital staff and avert negative repercussions, patients have described using drugs in their hospital rooms and patient washrooms, in other areas of the hospital, and in other nearby public locations [21, 22]. Patients often consume drugs in non-sterile locations, reuse syringes, and rush their injection. These actions only compound the risk of developing infections and other health complications [17, 21, 22]. Patients who continue to use drugs while hospitalized are also at increased risk of experiencing stigma [23, 24] and initiating discharge prior to treatment completion, and experiencing unplanned readmission and mortality [19, 25–27].

Extant research that explores VAD injection primarily describes the prospective and retrospective experiences of outpatients participating in this practice and suggests that the practice is uncommon. For example, of 33 PWID eligible to receive outpatient parenteral antimicrobial therapy within a residential addiction treatment facility, over 90% reported that their PICC line did not precipitate a desire to inject into the device or increase their motivation to use drugs [20]. In a cohort study of 67 PWID receiving outpatient therapy, only 2% of those who failed treatment did so because of VAD injection [12]. Three case studies have similarly described non-hospitalized people injecting into VADs, including a cannula self-inserted into a femoral vein [28], a central venous catheter [29], and a port-a-cath [30]. A recent Canadian qualitative study [31] examined the practice of injecting into PICC lines, a common type of vascular access device. Through retrospective interviews with 24 people who use drugs living with HIV/HCV who had been hospitalized at least once in the past 5 years and 26 healthcare providers, the authors found that even though few participants reported engaging in this practice, healthcare providers reported that fears of PICC line tampering influenced clinical care decision-making. Our study extends this work by exploring in depth the perspectives and experiences of VAD injection among a sample of PWID hospitalized on medical or surgical units of a large tertiary care facility.

### Objectives

The objectives of this analysis were to (1) describe inpatient experiences and motivations to inject drugs and/or diverted medications into VADs, and (2) propose clinically relevant and patient-centered recommendations to reduce the harms associated with this practice.

## Methods

### Setting

The study was completed at a large, urban acute care hospital in Western Canada that treats many structurally vulnerable patients with complex health and social needs and high rates of drug and alcohol use disorders. The hospital has an addiction medicine consultation team. At the time of this study, the consultation team offered patients with drug and alcohol use disorders expert pain and withdrawal management, opioid agonist and other medication treatment, harm reduction supplies, addiction counselling, and wraparound health and social supports (e.g., brokered access to housing and income supports, and health promotion interventions such as screening for sexually transmitted infections and immunizations) [24]. This study was conducted by a University-based researchers affiliated and colocated on site with the addiction medicine consultation team (KD is also medical director of the team).

### Methodology

Our findings are part of a broader mixed-method evaluation examining the provision of sterile injection supplies by the consultation team to hospital inpatients. The results described in this study organically emerged as the study progressed and were analyzed as an independent theme. We used a focused ethnography as a research approach, which is a time-limited method of eliciting detailed answers to delineated research questions within a distinct group or context, and semi-structured interviews as our sole data collection tool, which comprise predetermined open-ended questions. In line with previous applied health research studies, our focused ethnography did not include participant observation [32, 33]. The interview questions explored hospitalized participants’ experiences receiving clinical care, using drugs while hospitalized (including their perspectives of receiving sterile supplies), and their thoughts on how care for hospitalized PWID can be improved. A trained qualitative researcher (HB) obtained informed consent, audio-recorded, and conducted all interviews in a location within the hospital of the participants’ choosing. Participants received $20 CAD honoraria.

### Participant recruitment and data collection

Between April 20, 2017, and March 7, 2018, we approached inpatients from general medical and surgery units who had reported recent injection drug use to the consultation team and offered participation in a one-hour interview. We completed interviews with 25 patients (Table 1); theoretical saturation was reached after interview 21, meaning that no new concepts or themes emerge in subsequent interviews [32]. We asked almost all the participants (*n* = 24) about their perspectives of injecting drugs and/or diverted medications into VADs; one interview was terminated early by participant request.

**Table 1:** Participant information

Variable	Descriptive statistics *n* (%)
Demographics	*N* = 25
**Gender**	
Female	12 (48)
Male	12 (48)
Transgender	1 (4)
**Ethnicity**	
First Nations, Inuit, or Metis	20 (80)
White	5 (20)
**Age**	
30–39	7 (28)
40–49	10 (40)
50–59	7 (28)
60+	1 (4)

Drug use characteristics	*N* = 25
**Length of drug use (years)**	
1–10	13 (52)
11–20	4 (16)
21–30	3 (12)
31–40	5 (2)
**Utilization of drug consumption supplies**	
Accepted and used supplies	19 (76)
Did not accept supplies	4 (16)
Accepted but did not use supplies	2 (8)

### Data analysis

Interview recordings were transcribed and deidentified, and participants assigned pseudonyms. The average interview length was 51 min. The software ATLAS.ti 8 was used to organize the data iteratively. We employed latent content analysis, which entails examining, highlighting, and labeling groups of words and sorting the labeled text using codes that reflect similar meanings. This form of analysis is inductive allowing classifications to flow from the text [34]. We then explored latent aspects of the text by collating codes, collapsing and reconsidering categories, and then abstracting at a higher level of interpretation [34].

### Rigor

Rigor in qualitative research is a set of strategies used to strengthen study quality [32]. To ensure rigor, we employed a second experienced qualitative researcher who was also a member of the research team to randomly and separately analyze 20% of the transcripts to ensure concordance of interpretation [35]. Additionally, we regularly consulted members of an advisory group to elicit feedback on the appropriateness of study procedures and validity of the analysis. The advisory group comprised people with lived experience of drug use and hospitalization; over half also self-identified as Indigenous [35].

## Results

Nearly all participants had heard of patients injecting into their VADs (specifically PICC and IV lines). Participants implied that hospitalized PWID with VAD would have at least contemplated participating in the practice. Some participants had unsuccessfully attempted to inject into their VADs, but a few had successfully injected into their VADs at least once during their current hospitalization, or during previous hospitalizations.

### “You just connect her and…dial in direct”: reasons for injecting into a VAD

The primary motivation reported for this practice was easier venous access, because many participants and their peers had vein damage associated with long-term injection drug use or improper injection techniques. Injecting into their VADs was seen as “quicker,” “easy,” and “convenient” for people whose “veins [were] …all done.” Participants perceived injecting into a VAD as less harmful than prolonged attempts to secure venous access because it reduced the frequency of injection site injury and associated infection risks. Participants also described that acute withdrawal could complicate injection drug use and challenge venous access, further increasing the appeal of injecting into a VAD. As “Allison” told us:

‘Oh my god, some mornings, when it’s my first fix and I can’t do it and I just want to cry or just want to scream. It sometimes, it actually brings me to the point of tears because I can’t get it…It’s frustrating…It’s easier to [inject in your VAD] than having to find a vein and chance missing it. If you miss it then there goes the first shot you know. The strongest shot. The shot that counts.

For some participants, the frustration associated with finding venous access was so severe that they described wanting a dedicated VAD for drug use while in hospital and even to be discharged from hospital care with their VAD intact.

Well, they’re always going to use [their VAD] because you know, they don’t have to dig in their skin or anything, it’s a perfect little port. And you know, when a person is leaving [hospital] they’re all like, no you can’t leave with that. Let them, you’re only helping so that they’re not going to go digging around in their skin and stuff. - ‘Carl’

A few participants described how the psychoactive effects of drugs were stronger when using a VAD compared to injecting intravenously and believed some people may prefer injecting via this route.

I’ve done it. Yeah…It’s just an easy port...you don’t have to mark yourself up, you don’t have to look for nothing, you just connect her and...dial in direct...I think it just hits you a little bit harder and faster...somebody that wants to get high, yeah, it’s probably the more enticing route to go. - ‘Kenneth’

### “One awful, fuckin’ big chance”: awareness of potential risks

Participants who actively injected into VADs, or those who supported the practice, either described no associated risks or implied that the benefits of injecting outweighed the risks. Yet most refrained entirely from the practice or reported stopping when they learned of potential risks or experienced related harm. These participants perceived the practice as “scary,” “risky,” “dangerous,” and “one awful, fuckin’ big chance” and people who participate in it as “nuts.”

In terms of potential risks, participants worried that injecting into a VAD with non-sterile syringes or contaminated drugs could lead to circulatory issues or additional infection risks.

Oh yeah for sure. Right in the PICC line, for sure. It’s easy. It’s a mainline right to your veins. But that’s dangerous man, infection. Oh my god, lot of things, not good, I wouldn’t do it. I wouldn’t do it. I’m too scared. - ‘Leah’

In the process of diluting your substance...you might not even be able to see it but any particle...can be built right into that line, can get in your bloodstream and cause a clot, cause any kind of numerous problems, it’s very dangerous. - ‘Kenneth’

Many participants were also aware that some types of VADs provide more direct access to central veins and, as such, believed that injecting into these devices (i.e., PICC lines) may increase their risk of overdose. For example, “Silvia” described contemplating injecting into her PICC line but ultimately refrained. According to her, “I was actually thinking of it too, but then I thought… no, that’s a little bit too close to my heart.” “Silvia” did, however, describe a desire to inject into a VAD that was not a PICC line because of her struggles to inject herself. “Silvia’s” first experience with injecting drugs was when she was thirteen years old and after thirty years of use, she had developed severe venous damage. According to “Silvia”:

Like we could put it in there and then you flush it and then you lock it...Make it safer...I’m not saying PICC line site either. But like where it won’t fall out...people are not getting their shot, because they’re wasting it, because they can’t get a line.

One participant described overdosing after injecting into her VAD. When we asked “Elsa” if she had ever injected into a VAD, “Elsa” reported doing so a few times in the past. According to “Elsa,” “I’ll never do that again, because it goes directly to your heart and I like, I almost died.” “Elsa” described recent engagements with local harm reduction organizations where she received safer drug use education, and she had also been prescribed methadone. These supports, along with a personal desire to reduce physical harms to herself, helped “Elsa” refrain drug use during her current hospitalization.

Most participants, nevertheless, used general and vague terms when describing VADs and displayed limited knowledge of how the devices functioned. Several participants were not aware of how to inject properly into their VADs, despite the exigency some felt to use their devices.

I’ve heard people say they’ve done it but I don’t know how they could do the PICC line because it’s so long right. It goes all the way to…I don’t know, I would have to ask somebody to show me how they do it. - ‘Linda’

But I actually tried, that wouldn’t work as far as here, so I maybe take this thing off and put it back on after. When you do the flush it’s massive, then I, lose a lot of water, probably work too. I did almost try it but not really. Because I have such a hard time hitting myself. I thought it’d be easier, you know what I mean. - ‘Owen’

In addition, some participants reported unsubstantiated beliefs regarding VADs and safety. One participant implied that the risk of developing an infection after injecting into an IV line was less than the risk associated with injecting into a PICC line. According to “Andria,” “Obviously, going into the PICC line isn’t safe…but if they had like something in their foot…a foot you can lose, your heart…” Participants also described witnessing specific unsafe practices related to VADs. “Thomas” recounted witnessing a fellow patient “take a needle out of their leg” to utilize it for injecting drugs through other means. “Oliver” described patients injecting into their VAD to “flush syringes,” meaning to retract blood into a used syringe and then reinject, to ensure any leftover drug residue in the syringe was consumed. “Washing” of syringes, filters, and other injection drug equipment in this manner is a strategy used by PWID to stretch limited personal drug supplies [36], which may be more difficult to maintain while hospitalized and unable to participate in work to generate income.

### “Show them the process…to be as clean as possible”: reducing harms

Some participants we spoke with reported attempts to lower potential risks associated with injecting in their VADs by titrating their dose (using a small amount of drug to start) and keeping their VAD as sterile as possible. According to “Dean”:

Mm-hmm. I’ve done it… It gets better, you don’t miss or nothing, it’s already there….And it’s safe to do it as long you’re not doing a whole lot, you keep it clean like the nurses do, you clean it with swabs and that.

One participant felt most patients passively relied on nurses to keep their VAD clean. “Rhonda” explained that patients who inject in their VADs think to themselves, “I’ve got an IV, I don’t even have to try and find a vein now…the nurses come and flush them out before they do everything, so it stays clean.” However, “Andria” felt that staff should proactively educate patients with VADs on the risks associated with injecting drugs into their devices but also provide sterile injection supplies and knowledge on how to clean and maintain these devices if they choose to inject.

Maybe show them properly. Say show them how to use the flush. Show them to, say if you’re going to do it and you’re going to do it here, then you’re going to do it properly… have to set your own standards too, to what you feel is safe…you could educate them…and then show them the process…to be as clean as possible. - ‘Andria’

Yet only one participant described hospital staff discussing the practice of injecting into VADs. According to “Candice,” “the IV team, or the infectious disease team told me if I use my PICC line to get high they will shoot antibiotics into my [arm], muscular injection, the whole time I’m here.” She interpreted this admonition as benevolent concern on the part of her care team, and it successfully deterred her from using her device.

However, for most participants, the anticipation of severe repercussions, such as changes to their pain or withdrawal management medication regimes, resulted in them being mistrustful of hospital staff. They therefore concealed their drug use, including use that involved injecting into their VADs. “Curtis” considered injecting into his VAD, but according to him, “I didn’t, I didn’t, no I didn’t want to make it that noticeable that I was using when I left so to speak. When I had come back in, you can pretty much, [the nurses] know, you can just tell they know.” According to “Lydia,” nursing staff had suspected that she was actively consuming drugs after finding a powder in her purse and as a result, her pain medication regime was changed from tablet to liquid, which caused her distress. When “Lydia” was asked how to make sure patients were safe when injecting into their VAD, she responded that attempts by hospital staff to prevent drug use fostered mistrust and impeded patients from using safer injection techniques. According to “Lydia”:

Well, that’s when you explain to them about [using sterile needles]. But then when you have your nurses screwing that up for you guys, good luck. Of course they’re going to go back to hiding, right?...People would care about having [sterile needles] and doing it cleanly and dadadada if, you know, you weren’t gonna get in trouble, right?

## Discussion

In this study, nearly all participants were aware of the practice of injecting drugs into VAD. However, most participants chose not to regularly inject into their VAD because they perceived the practice as risky. Of those who participated in the practice, most described easier venous access as the primary motivation for themselves and others to inject into their VADs. This practice was often seen as pragmatic and less deleterious than direct intravenous injection, particularly for people who had difficulty finding a vein and injecting themselves. Despite these reported advantages, many participants described a lack of knowledge regarding how VADs functioned. Several participants, including some who had injected into their VADs, appeared unaware of the potential negative health outcomes or strategies to reduce associated risks and implied a need for nonjudgmental education to ensure their safety while injecting into their VADs.

Injecting drugs into VADs is a concern for clinicians administering parenteral antimicrobial therapy to PWID and is often the primary reason PWID are deemed ineligible for outpatient treatment [9–11]. Inpatient supervision by hospital staff is advised to prevent this practice [9–11], but our findings suggest that this advice may not be effective at either informing patients of the risks of, or preventing, VAD injecting. Moreover, extant literature indicates that other common interventions designed to deter in-hospital drug use (e.g., confiscation of injection supplies, enhanced surveillance, and monitoring) may in fact increase risks for patients (e.g., rushed injection, using alone and in unsafe circumstances, premature discharge) [21, 22]. Our study did not systematically explore whether staff took measures to deter non-medical VAD nor whether participants experienced harms because of these deterrence measures. However, participant accounts demonstrated a need for hospital staff to be aware of the motivations and experiences of PWID injecting in their VADs. This may aid in providers anticipating and addressing patient needs through non-stigmatizing care, which could include staff adopting trauma-informed approaches to patient care, being mindful of words and body language when interacting with PWID, and prioritizing patient autonomy and choice [37, 38].

A key strategy for addressing VAD injecting is to reduce hospitalized patients’ need to inject drugs in the first place. It is imperative that patients have expeditious access to effective, tailored pain and withdrawal management [39, 40], injectable and oral opioid agonist therapy [41, 42], counselling [39, 42], and social supports, including peer support and social workers [24, 43] during their hospitalization. These interventions should be provided with the active consent and collaboration of patients.

There is also some evidence to suggest positive treatment outcomes in PWID receiving parenteral antimicrobial therapy outside of acute care hospitals [13, 14, 44, 45]. Patients with current or previous injection drug use have successfully completed parenteral therapy and participated in minimal, or at least comparable to average, drug use while residing in medical respite facilities (i.e., temporary shelters that provide medical services to people experiencing homelessness) [13, 46], and from within their private homes [44]. With sufficient support (i.e., access to stable housing, substance use disorder discharge planning, regular check-ins with clinical staff, access to agonist medication or other substance use treatment), PWID may be able to successfully complete parenteral antimicrobial therapy in the community and have low rates of substance use and VAD injection [13, 14, 17, 44, 45].

It is also important to recognize that even with maximal medical and social support, some PWID will continue to inject while hospitalized. For example, empirical evidence suggests that prolonged drug use may result in impaired volitional control [47]. Additionally, there are currently no evidence-based pharmaceutical treatments for those with stimulant use disorders [48] and many acute care facilities still do not provide patients with opioid use disorders evidence-based medication treatment or harm reduction interventions [49]. Clinicians therefore should be educated and encouraged to engage patients with previous or active injection drug use in non-stigmatizing and factual conversations about reducing harms associated with drug use, including the potential risks of injecting into VADs [15, 50]. Research to quantify VAD injecting risks is urgently needed to facilitate such factual discussions. Meanwhile, staff may consider periodic laboratory monitoring to identify and treat incipient infections due to drug use [17]. Several outpatient clinics have established patient care plans, which have included verbal or written agreements from patients to refrain from injecting into their VADs [13, 14, 51, 52]. Clinics have also utilized specialized dressing or security seals on patients’ VADs to more easily detect drug use [13, 14, 51, 52]. However, these technologies were not employed in our study setting, and patients’ perspectives of these preventive measures are currently unknown, as is their effectiveness for preventing harm to patients.

If injection drug use into VADs is suspected or confirmed, discharging patients or abruptly discontinuing or modifying antimicrobial therapy to a less appropriate regimen is not recommended [9, 22, 53, 54]. These actions may preclude patients from completing vital treatment, compound the burden on hospital staff by increasing the likelihood of patients experiencing unplanned readmissions [54], and increase patients’ risk of mortality [27, 55]. Instead, hospital policies should address how patients who continue to use drugs will be supported to complete their medical treatment [38]. Staff should be trained and encouraged to have non-stigmatizing and transparent conversations with patients and offer patients sterile injection supplies, safe syringe disposal instructions, and a naloxone kit [53]. To obviate patients' perceived need to inject into their VADs, clinical staff or a peer support worker could also educate patients on vein finding and maintenance to encourage safe injection practices [56, 57].

A hospital-based supervised consumption service, if available [58, 59], could further reduce health risks of VAD injecting. Supervised consumption services are well described in the literature [60, 61] and aim to provide a safer and cleaner environment where people can consume pre-obtained drugs in hospital under the supervision of trained staff without the need to rush or fear of criminal prosecution [59]. Supports available within these services, such as nursing assistance to locate a vein, may result in fewer patients needing to use their VAD due to inability to find other venous access. Early experience suggests that when patients are given access to supervised consumption services in hospital settings, the incidence of injecting into VAD is quite low (i.e., occurring in only 5% of visits) [58]. Patients with refractory opioid use disorders could also be prescribed injectable opioid agonist therapy (i.e., hydromorphone, diacetylmorphine) and receive their doses (via self-injection or nurse-administered intramuscular injection) within the supervised consumption service. Injectable opioid agonist therapy is well established in community settings and has been shown to reduce drug use and improve treatment retention [62, 63].

In cases where education and available supports have not deterred VAD injecting, staff might consider supervising patients injecting into their VADs from within their hospital room (in jurisdictions where this is permissible) [64], or demonstrating how to more safely and sterilely inject into VADs, requesting patients avoid certain VADs, and establishing a non-punitive system for patients to report to hospital staff after use [15, 57]. However, more research is needed to evaluate the effectiveness of these harm reduction strategies for reducing health harms of VAD injecting for hospitalized PWID.

## Strengths and limitations

This study supports recent research that suggests hospital policies regarding the use of PICC lines in hospitalized PWID are inadequate and that the integration of harm reduction strategies into clinical care is needed [31]. However, this study used a subset of data from a broader mixed-method evaluation that elicited the perspectives of inpatients who reported recent or active injection drug use to a harm reduction addiction medicine consultation team and who were offered sterile injection supplies at the bedside (most participants accepted those supplies). As such, awareness of and experience with VAD injection may have been higher than other hospitalized patients with a history of drug use. Also, use of VADs for injection emerged as an independent theme as the study progressed, limiting a more in-depth a priori exploration of this topic. Finally, this study was conducted in a large city in Western Canada and may not be generalizable to other dissimilar contexts.

## Conclusions

Given the imperative to ensure PWID receive high-quality hospital care and are able to complete their antimicrobial treatments, we have outlined several potential strategies that could help to address the issue of VAD injecting in hospitals. However, further research is needed to identify and quantify the actual health risks and prevalence of this practice. Quantitative and qualitative studies that elicit the perspectives of both clinical staff and PWID could determine the acceptability and optimal mix of interventions for preventing potentially deleterious patient outcomes associated with injecting into VADs.

## Data Availability

Data sharing is not applicable to this article because it is a qualitative study.

## Abbreviations

IV: Intravenous line
PICC: Peripherally inserted central catheter
PWID: People who inject drugs
VAD: Vascular access device
